# Enhancing nutrition education resources through the development and refinement of a checklist using the suitability assessment of materials (SAM)

**DOI:** 10.1177/02601060251365357

**Published:** 2025-08-17

**Authors:** Oliver Sage, Flora Wang, Chiara DiAngelo, Sandra Marsden, Claudia Faustini, Shannan Grant, Tamara R Cohen

**Affiliations:** 1 Faculty of Land and Food Systems, Food Nutrition and Health, 8166 The University of British Columbia, Vancouver, BC, Canada; 2 Canadian Sugar Institute, Toronto, ON, Canada; 3 Department of Mathematics and Statistics, 5618 Concordia University, Montréal, QC, Canada; 4 Department of Applied Human Nutrition, Faculty of Professional Studies, Mount Saint Vincent University, Halifax, NS, Canada

**Keywords:** Suitability, nutrition education, resources, tools, registered dietitian

## Abstract

**Background:**

Evidence-based nutrition education resources are one way to help registered dietitians (RDs) translate scientific knowledge to consumers.

**Aim:**

To develop a checklist based on suitability assessment of materials (SAM) and to assess its use to refine nutrition education resources.

**Methods:**

RDs were recruited online to assess two nutrition education resources using SAM. Three rounds of surveying and two rounds of resource refinements occurred. A “checklist” was created to refine the resources between rounds. Descriptive statistics and nonparametric tests were performed to explore differences in SAM-scores between rounds.

**Results:**

RDs participated in the first (*n* = 45), second (*n* = 37), and third (*n* = 27) surveys. SAM-scores significantly improved in both resources by the third round. The refined checklist included more explicit instructions and provided examples to help guide resource changes.

**Conclusions:**

Using the checklist improved SAM scores. Future work should include end-users to help with checklist validation.

## Introduction

Registered dietitians (RDs) are health professionals who use nutrition education resources, such as fact sheets, to communicate scientific information and support client understanding. To ensure these materials are effective, they must be appropriate for the intended audience. The suitability assessment of materials (SAM), by Doak, Doak, and Root in 1996, is a tool used to evaluate patient-education materials (Doak et al., 1996). It assesses these materials based on several key factors: content clarity and focus, literacy demand aiming for a fifth-grade reading level or lower, quality and relevance of graphics, layout and typography, learning stimulation and motivation, and cultural appropriateness. By considering these aspects, SAM ensures that educational resources are accessible, understandable, and effective for diverse audiences. Research has shown that the SAM tool demonstrates acceptable internal consistency, with a Cronbach’s alpha coefficient of 0.75 (Anderson et al., 2014).

Despite its utility, SAM is often used only as an evaluation tool, with limited guidance on how to revise materials once deficiencies are identified. This creates a gap between assessment and actionable improvement. To our knowledge, no formal, dietitian-developed checklist exists to help systematically revise nutrition education resources based on SAM findings. The objectives of this project were to develop a checklist based on SAM and assess its use to refine nutrition education resources originally developed in 2016 by the Canadian Sugar Institute.

## Methods

This is a mixed-methods instrument development and evaluation study. Participants were English-speaking Canadian RDs who were recruited from social media and listservs between August 2022 and October 2023. After consenting, RDs completed an online demographic questionnaire through Qualtrics ^®^ (Provo, UT). They evaluated two nutrition education resources (Supplemental Figure 1) that were originally developed in 2016 on the topic of sugar and health using SAM, which asks about (a) content, (b) literacy demand, (c) numeracy, (d) graphics, (e) layout/typography, and (f) learning stimulation/motivation using a 3-point Likert Scale 0 (not suitable), 1 (adequate), or 2 (superior). The RDs were informed that the resources were intended for use by dietitians. The co-authors did not independently assess the tools using the SAM instrument prior to the study's launch.

### Checklist development and tool refinement

After the first round (September to December 2022) (*n* = 45), data was analyzed and a checklist was developed by two researchers based on the components of SAM, adopted from Smith et al. (Smith et al., 2014) to ensure relevance for health-professional related resources. CAD (a RD not involved in the study) then used the checklist to refine the tools (Supplemental Figure 2). The same RDs were asked to re-score [(January to March 2023) Round 2, *n* = 37] the refined tools using SAM and the checklist was refined again based on SAM scores to improve its use. Specifically, the checklist was refined to improve organization and user-friendliness. Items were grouped into categories, and checkboxes were added to allow users to track their progress. Notes sections were added to document how each item was addressed or needed improvement. Finally, a section on “Target Audience and Branding” was also introduced, responding to feedback from the dietitians about the importance of understanding the intended audience and ensuring consistent branding throughout.

The results were analyzed again and CAD refined the tools again using the refined checklist (Version 2.0). A third survey [(June to October 2023) *n* = 27 RDs, 3 of which were “new”] was launched and CAD refined the tools one last time, at which point the checklist was refined and finalized (Figure 1). Specifically, the finalized checklist included the following updates: the addition of logos and branding. The notes sections were removed following this round of testing, as they were deemed unnecessary and made the resource overly lengthy; this adjustment streamlined the checklist to a concise two-page format for ease of use.

**Figure 1.**
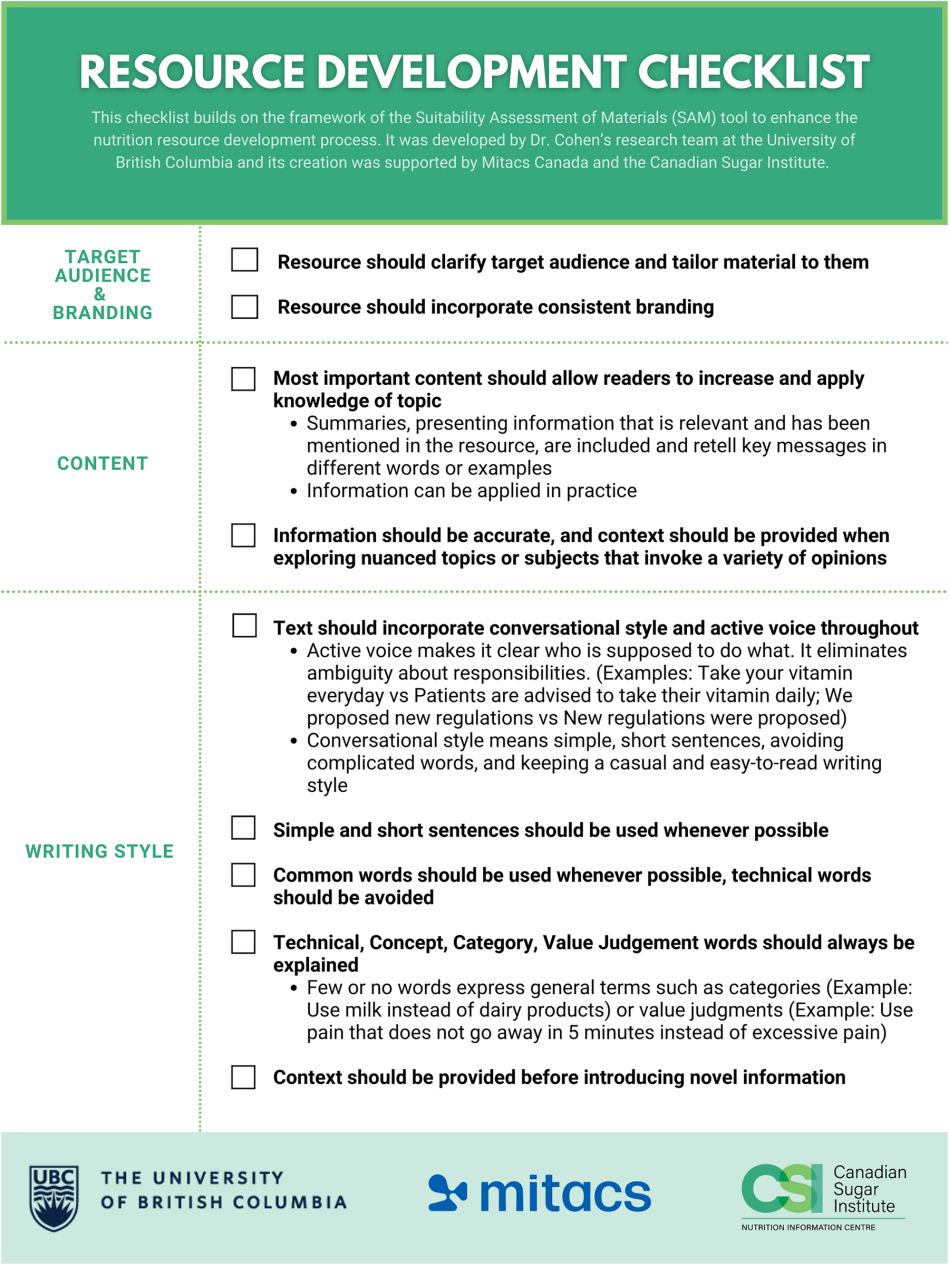
Resource Checklist.

**Figure 1.**
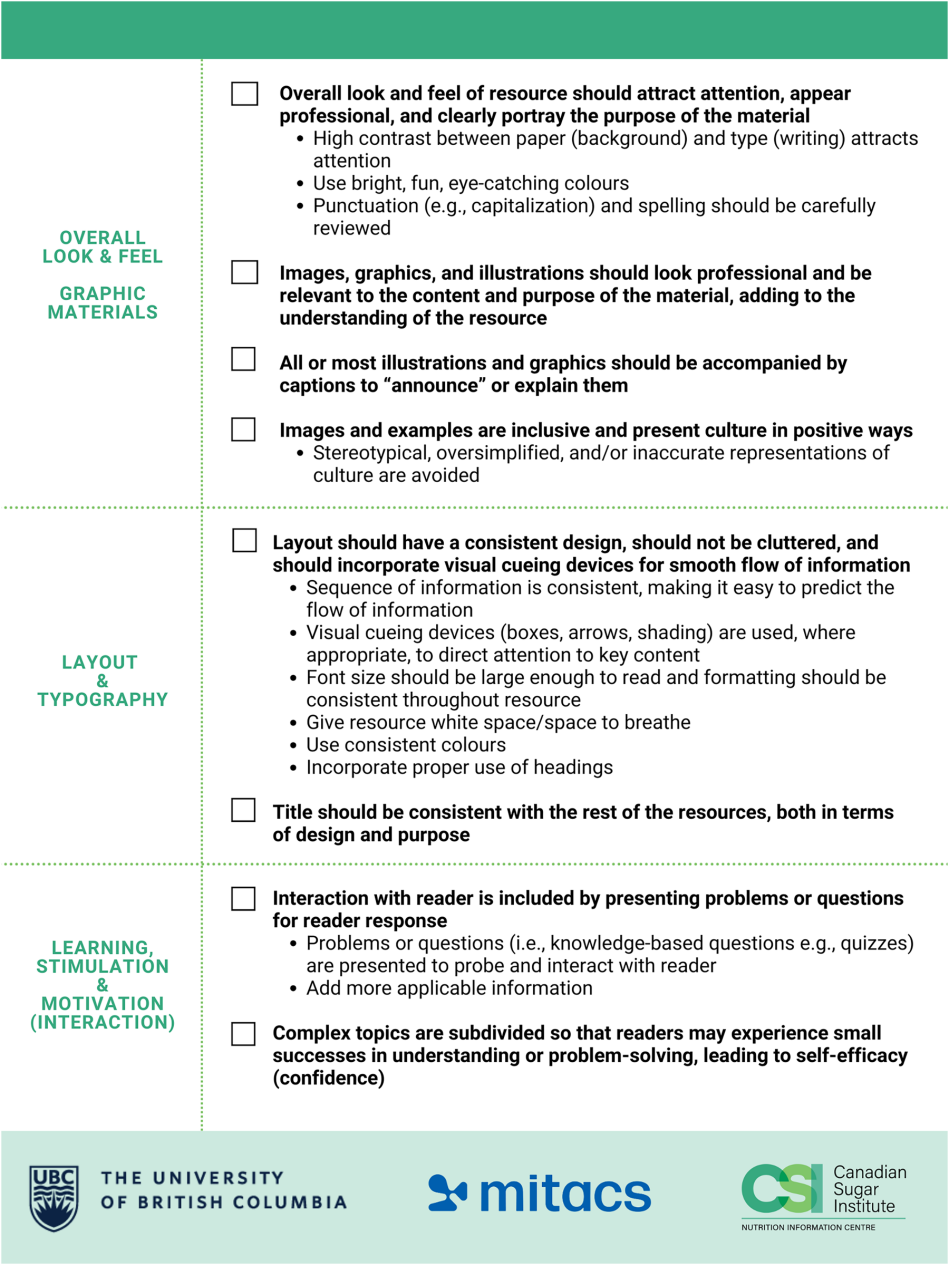


At the end of Survey 3, RDs were asked whether they had participated in the previous surveys and whether they believed the refined resources were improvements on the original resources (yes/no).

### Statistical analysis

Analyses were conducted in SAS^®^ Studio (version 3.81). Descriptive statistics were used to summarize participant characteristics, and SAM scores. Next, the total SAM score was divided by the maximum total score and converted to a percentage to describe the scores as unsuitable (0–39%), adequate (40–69%), or superior (70–100%) [7]. SAM scores by category were analyzed separately whereby the scores were summed per category, and then divided them by the maximum SAM score and also converted these into percentages. Fisher's exact test of independence was used to determine the association between surveys and the SAM scores and Friedman's nonparametric test was used to assess for significance in the SAM score. Post-hoc analysis using the Dunn–Bonferroni method was conducted after Friedman's test to analyze the pairwise comparisons of the difference in means between any pair of surveys for which the means were significantly different. Finally, a Sensitivity Analysis using the Wilcoxon signed-rank test was conducted to assess for significant differences in results for Survey 3 with and without the *n* = 3 additional RDs; key findings did not differ therefore our results include those *n* = 3 RD. The statistical significance level was set at 0.05.

## Results

This study included mostly women (*n* = 44) who have been practicing dietetics for >2 years (Table 1). Eighty-nine percent of the participants in *Survey 3* had participated in the previous surveys.

**Table 1.** Study participant count and frequency [*n* (%)].

	Survey 1	Survey 2	Survey 3
Total number (*n*)	45	37	24^b^
Gender			
Women	44 (98%)	37 (100%)	23 (96%)
Men	1 (2%)	0 (0%)	0 (0%)
Unknown	0 (0%)	0 (0%)	1 (4%)
Area of practice^a^			
Outpatient care	17 (38%)	13 (35%)	8 (20%)
Private practice	13 (29%)	12 (32%)	7 (29%)
Long-term care	7 (15%)	6 (16%)	6 (25%)
Acute hospital	8 (17%)	6 (16%)	4 (17%)
Population and public health	8 (17%)	8 (22%)	2 (8%)
Food service management	1 (2%)	1 (3%)	0 (0%)
Sports nutrition	4 (2%)	4 (11%)	2 (8%)
Corporate wellness	3 (6%)	3 (8%)	2 (8%)
Industry	1 (2%)	1 (3%)	1 (4%)
Other	9 (20%)	8 (22%)	7 (29%)
Time working as a registered dietitian			
≤2 years	9 (20%)	8 (22%)	6 (25%)
2–5 years	18 (40%)	16 (43%)	10 (41%)
5–15 years	12 (26%)	6 (16%)	4 (17%)
15+ years	6 (13%)	7 (19%)	3 (12%)

^a^ Participants were given the option to select more than one area of practice. One participant in Survey 3 did not answer the question about time working as a registered dietitian.

^b^ The demographic information of the three new participants that were recruited in Survey 3 was not collected.

Table 2 displays the results of the SAM scores for each resource from each round. The total (mean ± standard deviation) SAM score of Resource 1 improved from 66.6 ± 16.0% (“adequate”) in Survey 1 to 78.8 ± 16.7% (“superior”) in Survey 3 (*p* = 0.007). Significant improvements were seen primarily for Writing Style 1 (*p* = 0.021), Vocabulary 3 (*p* = 0.024), Layout (*p* = 0.039) and Interaction (*p* < 0.0001). For Resource 2, total SAM scores significantly improved from *Survey 1* [70.6 ± 18.9% (“superior”)] to *Survey 3* [82.0 ± 16.6% (“superior”)] (*p* = 0.016), with most significant changes shown for the variables titled Quick Glance (*p* = 0.003), Types of Illustrations/Images (*p* = 0.008), Typography (*p* = 0.004), Interaction (*p* < 0.0001), and Cultural Images and Examples (*p* < 0.0001).

**Table 2.** SAM ratings [frequency (%) and total SAM scores (mean ± SD) for nutrition education resources from Survey 1 to Survey 3 [*n* (%)].^a^

Resource	Variables	Rating	Survey 1 (*n* = 45)	Survey 2 (*n* = 37)	Survey 3 (*n* = 27)	*P*-value
Resource 1 “Effects of Sugars on Metabolic Disease”	Content					
	*Purpose*	Superior Adequate Not suitable	24 (54.6) 19 (43.2) 1 (2.3)	25 (67.6) 12 (32.4) 0 (0)	21 (80.8) 5 (19.2) 0 (0)	0.130
	Mean SAM score		1.52 ± 0.54	1.68 ± 0.47	1.81 ± 0.40	0.072
	*Content Topics*	Superior Adequate Not suitable	20 (45.5) 23 (52.3) 1 (2.3)	19 (51.4) 18 (48.6) 0 (0)	18 (69.2) 8 (30.8) 0 (0)	0.228
	Mean SAM score		1.43 ± 0.55	1.51 ± 0.51	1.69 ± 0.47	0.138
	*Summary/Review*	Superior Adequate Not suitable	29 (65.9) 14 (31.8) 1 (2.3)	25 (67.6) 10 (27.0) 2 (5.4)	21 (80.8) 5 (19.2) 0 (0)	0.628
	Mean SAM score		1.64 ± 0.53	1.62 ± 0.59	1.81 ± 0.40	0.364
	Literacy Demand					
	*Reading Grade Level*	Superior Adequate Not suitable	43 (95.6) 2 (4.4) 0 (0)	35 (94.6) 0 (0) 2 (5.4)	25 (92.6) 1 (3.7) 1 (3.7)	0.36
	Mean SAM score		1.96 ± 0.21	1.89 ± 0.46	1.89 ± 0.42	0.858
	*Writing Style 1*	Superior Adequate Not suitable	23 (56.1) 8 (19.5) 10 (24.4)	15 (41.7) 17 (47.2) 4 (11.1)	20 (80.0) 4 (16.0) 1 (4.0)	0.005
	Mean SAM score		1.31 ± 0.85	1.31 ± 0.67	1.76 ± 0.52^c^	0.021
	*Writing Style 2*	Superior Adequate Not suitable	11 (25.0) 20 (45.5) 13 (29.5)	13 (37.1) 17 (48.6) 5 (14.3)	15 (55.6) 3 (11.1) 9 (33.3)	0.004
	Mean SAM score		0.95 ± 0.75	1.23 ± 0.69	1.22 ± 0.93	0.193
	*Vocabulary 1*	Superior Adequate Not suitable	13 (29.5) 23 (52.3) 8 (18.2)	7 (19.4) 25 (69.4) 4 (11.1)	12 (44.4) 11 (40.7) 4 (14.8)	0.183
	Mean SAM score		1.11 ± 0.69	1.08 ± 0.55	1.30 ± 0.72	0.337
	*Vocabulary 2*	Superior Adequate Not suitable	13 (32.5) 23 (57.5) 4 (10.0)	16 (48.5) 17 (51.5) 0 (0)	14 (56.0) 9 (36.0) 2 (8.0)	0.113
	Mean SAM score		1.23 ± 0.62	1.48 ± 0.51	1.48 ± 0.65	0.121
	*Vocabulary 3*	Superior Adequate Not suitable	11 (30.6) 18 (50.0) 7 (19.4)	10 (38.5) 15 (57.7) 1 (3.9)	15 (68.2) 5 (22.7) 2 (9.1)	0.022
	Mean SAM score		1.11 ± 0.71	1.35 ± 0.56	1.59 ± 0.67^b^	0.024
	*Sentence Construction*	Superior Adequate Not suitable	22 (52.4) 18 (42.9) 2 (4.8)	25 (71.4) 10 (28.6) 0 (0)	20 (76.9) 6 (23.1) 0 (0)	0.142
	Mean SAM score		1.48 ± 0.59	1.71 ± 0.46	1.77 ± 0.43	0.058
	Graphic Material					
	*Quick Glance*	Superior Adequate Not suitable	9 (20.0) 30 (66.7) 6 (13.3)	9 (24.3) 23 (62.2) 5 (13.5)	11 (40.7) 14 (51.9) 2 (7.4)	0.425
	Mean SAM score		1.07 ± 0.58	1.11 ± 0.61	1.33 ± 0.62	0.161
	*Types of Illustrations/Images*	Superior Adequate Not suitable	33 (73.3) 12 (26.7) 0 (0)	29 (78.4) 7 (18.9) 1 (2.7)	17 (63.0) 10 (37.0) 0 (0)	0.317
	Mean SAM score		1.73 ± 0.45	1.76 ± 0.49	1.63 ± 0.49	0.432
	*Relevance of Images*	Superior Adequate Not suitable	23 (51.1) 21 (46.7) 1 (2.2)	21 (56.8) 14 (37.8) 2 (5.4)	16 (59.3) 11 (40.7) 0 (0)	0.780
	Mean SAM score		1.49 ± 0.55	1.51 ± 0.61	1.59 ± 0.50	0.767
	*Graphics*	Superior Adequate Not suitable	35 (87.5) 5 (12.5) 0 (0)	29 (82.9) 6 (17.1) 0 (0)	23 (88.5) 3 (11.5) 0 (0)	0.816
	Mean SAM score		1.88 ± 0.33	1.83 ± 0.38	1.88 ± 0.33	0.783
	Layout & Typography					
	*Learning Enhancement (road signs)*	Superior Adequate Not suitable	30 (68.2) 13 (29.6) 1 (2.3)	29 (78.4) 8 (21.6) 0 (0)	23 (85.2) 3 (11.1) 1 (3.7)	0.260
	Mean SAM score		1.66 ± 0.53	1.78 ± 0.42	1.81 ± 0.48	0.265
	*Typography*	Superior Adequate Not suitable	34 (77.3) 7 (15.9) 3 (6.8)	26 (72.2) 8 (22.2) 2 (5.6)	21 (77.8) 5 (18.5) 1 (3.7)	0.955
	Mean SAM score		1.70 ± 0.59	1.67 ± 0.59	1.74 ± 0.53	0.851
	*Captions*	Superior Adequate Not suitable	23 (52.3) 13 (29.6) 8 (18.2)	24 (68.6) 8 (22.9) 3 (8.6)	14 (58.3) 9 (37.5) 1 (4.2)	0.340
	Mean SAM score		1.34 ± 0.78	1.60 ± 0.65	1.54 ± 0.59	0.273
	*Layout*	Superior Adequate Not suitable	21 (46.7) 18 (40.0) 6 (13.3)	19 (51.4) 15 (40.5) 3 (8.1)	20 (76.9) 5 (19.2) 1 (3.9)	0.143
	Mean SAM score		1.33 ± 0.71	1.43 ± 0.65	1.73 ± 0.53^b^	0.039
	Learning, Stimulation, Motivation					
	*Interaction*	Superior Adequate Not suitable	3 (7.0) 4 (9.3) 36 (83.7)	8 (22.9) 16 (45.7) 11 (31.4)	8 (30.8) 15 (57.7) 3 (11.5)	<0.0001
	Mean SAM score		0.23 ± 0.57	0.91 ± 0.74^b^	1.19 ± 0.63^b^	<0.0001
	*Motivation*	Superior Adequate Not suitable	21 (50.0) 20 (47.6) 1 (2.4)	22 (61.1) 14 (38.9) 0 (0)	21 (77.8) 6 (22.2) 0 (0)	0.104
	Mean SAM score		1.48 ± 0.55	1.61 ± 0.49	1.78 ± 0.42	0.064
	Suitability					
	*Suitability for Population*	Superior Adequate Not suitable	28 (62.2) 10 (22.2) 7 (15.6)	27 (77.1) 4 (11.4) 4 (11.4)	21 (77.8) 4 (14.8) 2 (7.4)	0.569
	Mean SAM score		1.47 ± 0.76	1.66 ± 0.68	1.70 ± 0.61	0.248
	Total SAM Score (%) (mean ± SD)		66.6 ± 16.0	72.7 ± 14.9	78.8 ± 16.7^b^	0.007
Resource 2 “Frequently Asked Questions: Sources of Sucrose”	Content					
	*Purpose*	Superior Adequate Not suitable	39 (86.7) 5 (11.1) 1 (2.2)	30 (81.1) 7 (18.9) 0 (0)	22 (81.5) 4 (14.8) 1 (3.7)	0.710
	Mean SAM score		1.84 ± 0.42	1.81 ± 0.40	1.78 ± 0.51	0.773
	*Content Topics*	Superior Adequate Not suitable	32 (71.1) 13 (28.9) 0 (0)	26 (70.3) 11 (29.7) 0 (0)	23 (88.5) 3 (11.5) 0 (0)	0.179
	Mean SAM score		1.71 ± 0.46	1.70 ± 0.46	1.88 ± 0.33	0.193
	*Summary/Review*	Superior Adequate Not suitable	21 (47.7) 14 (31.8) 9 (20.5)	21 (56.8) 11 (29.7) 5 (13.5)	21 (77.8) 5 (18.5) 1 (3.7)	0.136
	Mean SAM score		1.27 ± 0.79	1.43 ± 0.73	1.74 ± 0.53^b^	0.031
	Literacy Demand					
	*Reading Grade Level*	Superior Adequate Not suitable	39 (86.7) 2 (4.4) 4 (8.9)	27 (73.0) 1 (2.7) 9 (24.3)	23 (85.2) 1 (3.7) 3 (11.1)	0.358
	Mean SAM score		1.78 ± 0.60	1.49 ± 0.87	1.74 ± 0.66	0.209
	*Writing Style 1*	Superior Adequate Not suitable	36 (81.8) 8 (18.2) 0 (0)	30 (85.7) 4 (11.4) 1 (2.9)	22 (91.7) 2 (8.3) 0 (0)	0.586
	Mean SAM score		1.82 ± 0.39	1.83 ± 0.45	1.92 ± 0.28	0.556
	*Writing Style 2*	Superior Adequate Not suitable	34 (77.3) 8 (18.2) 2 (4.6)	24 (66.7) 11 (30.6) 1 (2.8)	21 (84.0) 3 (12.0) 1 (4.0)	0.464
	Mean SAM score		1.73 ± 0.54	1.64 ± 0.54	1.80 ± 0.50	0.332
	*Vocabulary 1*	Superior Adequate Not suitable	23 (51.1) 20 (44.4) 2 (4.4)	18 (48.7) 18 (48.7) 1 (2.7)	19 (73.1) 7 (26.9) 0 (0)	0.261
	Mean SAM score		1.47 ± 0.59	1.46 ± 0.56	1.73 ± 0.45	0.105
	*Vocabulary 2*	Superior Adequate Not suitable	28 (66.7) 12 (28.6) 2 (4.8)	23 (69.7) 10 (30.3) 0 (0)	20 (83.3) 4 (16.7) 0 (0)	0.455
	Mean SAM score		1.62 ± 0.58	1.70 ± 0.47	1.83 ± 0.38	0.307
	*Vocabulary 3*	Superior Adequate Not suitable	32 (86.5) 3 (8.1) 2 (5.4)	21 (72.4) 8 (27.6) 0 (0)	20 (87.0) 2 (8.7) 1 (4.4)	0.125
	Mean SAM score		1.81 ± 0.52	1.72 ± 0.45	1.83 ± 0.49	0.339
	*Sentence Construction*	Superior Adequate Not suitable	31 (75.6) 8 (19.5) 2 (4.9)	25 (71.4) 10 (28.6) 0 (0)	22 (81.5) 5 (18.5) 0 (0)	0.538
	Mean SAM score		1.71 ± 0.56	1.71 ± 0.46	1.81 ± 0.40	0.664
	Graphic Material					
	*Quick Glance*	Superior Adequate Not suitable	21 (46.7) 16 (35.6) 8 (17.8)	16 (43.2) 20 (54.1) 1 (2.7)	21 (77.8) 6 (22.2) 0 (0)	0.003
	Mean SAM score		1.29 ± 0.76	1.41 ± 0.55	1.78 ± 0.42^b^^,^^c^	0.009
	*Types of Illustrations/Images*	Superior Adequate Not suitable	24 (54.6) 10 (22.7) 10 (22.7)	19 (54.3) 14 (40.0) 2 (5.7)	20 (83.3) 4 (16.7) 0 (0)	0.008
	Mean SAM score		1.32 ± 0.83	1.49 ± 0.61	1.83 ± 0.38^b^	0.023
	*Relevance of Images*	Superior Adequate Not suitable	14 (31.8) 27 (61.4) 3 (6.8)	13 (39.4) 19 (57.6) 1 (3.0)	16 (64.0) 7 (28.0) 2 (8.0)	0.054
	Mean SAM score		1.25 ± 0.58	1.36 ± 0.55	1.56 ± 0.65	0.070
	*Graphics*	Superior Adequate Not suitable	25 (71.4) 6 (17.1) 4 (11.4)	23 (76.7) 5 (16.7) 2 (6.7)	19 (82.6) 3 (13.0) 1 (4.4)	0.909
	Mean SAM score		1.60 ± 0.69	1.70 ± 0.60	1.78 ± 0.52	0.582
	Layout & Typography					
	*Learning Enhancement (road signs)*	Superior Adequate Not suitable	30 (68.2) 10 (22.7) 4 (9.1)	29 (78.4) 7 (18.9) 1 (2.7)	22 (81.5) 5 (18.5) 0 (0)	0.517
	Mean SAM score		1.59 ± 0.66	1.76 ± 0.49	1.81 ± 0.40	0.308
	*Typography*	Superior Adequate Not suitable	19 (43.2) 20 (45.5) 5 (11.4)	16 (59.3) 3 (11.1) 8 (29.6)	14 (66.7) 2 (9.5) 5 (23.8)	0.004
	Mean SAM score		1.32 ± 0.67	1.30 ± 0.91	1.43 ± 0.87	0.645
	*Captions*	Superior Adequate Not suitable	36 (83.7) 5 (11.6) 2 (4.7)	24 (66.7) 9 (25.0) 3 (8.3)	22 (81.5) 5 (18.5) 0 (0)	0.299
	Mean SAM score		1.79 ± 0.51	1.58 ± 0.65	1.81 ± 0.40	0.157
	*Layout*	Superior Adequate Not suitable	29 (64.4) 12 (26.7) 4 (8.9)	17 (47.2) 15 (41.7) 4 (11.1)	19 (70.4) 8 (29.6) 0 (0)	0.196
	Mean SAM score		1.56 ± 0.66	1.36 ± 0.68	1.70 ± 0.47	0.108
	Learning, Stimulation, Motivation					
	*Interaction*	Superior Adequate Not suitable	11 (25.6) 7 (16.3) 25 (58.1)	11 (29.7) 21 (56.8) 5 (13.5)	8 (29.6) 18 (66.7) 1 (3.7)	<0.0001
	Mean SAM score		0.67 ± 0.87	1.16 ± 0.65^b^	1.26 ± 0.53^b^	0.002
	*Motivation*	Superior Adequate Not suitable	22 (52.4) 19 (45.2) 1 (2.4)	22 (59.5) 13 (35.1) 2 (5.4)	18 (66.7) 9 (33.3) 0 (0)	0.644
	Mean SAM score		1.50 ± 0.55	1.54 ± 0.61	1.67 ± 0.48	0.483
	Suitability					
	*Cultural Images & Examples*	Superior Adequate Not suitable	3 (7.0) 17 (39.5) 23 (53.5)	12 (34.3) 21 (60.0) 2 (5.7)	16 (59.3) 10 (37.0) 1 (3.7)	<0.0001
	Mean SAM score		0.53 ± 0.63	1.29 ± 0.57^b^	1.56 ± 0.58^b^^,^^c^	<0.0001
	*Suitability for Population*	Superior Adequate Not suitable	22 (50.0) 13 (29.6) 9 (20.5)	23 (63.9) 7 (19.4) 6 (16.7)	20 (74.1) 5 (18.5) 2 (7.4)	0.342
	Mean SAM score		1.30 ± 0.79	1.47 ± 0.77	1.67 ± 0.62	0.115
	Total SAM Score (%) (mean ± SD)		70.6 ± 18.9	72.8 ± 16.8	82.0 ± 16.6^b^	0.016

Note: SAM: suitability assessment of materials; SD: standard deviation. SAM scores are graded as 0, 1, or 2, and total SAM score is reported as a percentage of points earned out of total possible points. *P < .05 considered statistically significant.

^a^ Friedman's nonparametric test comparing the mean scores of the three different surveys.

^b^ Post-hoc Dunn's test: means significantly different from Survey 1 when *P*-value is adjusted by the Bonferroni method.

^c^ Post-hoc Dunn's test: means significantly different from Survey 2 when *P*-value is adjusted by the Bonferroni method.

Writing Style 1: Use of conversational style and active voice.

Writing Style 2: Use of simple sentences.

Vocabulary 1: Use of common words.

Vocabulary 2: Use of technical, concept, category, value judgment words (CCVJ).

Vocabulary 3: Use of appropriate imagery words

Overall, 85.2% of the RDs ranked Resource 1 as having improved in comparison to the original version. One hundred percent of participants thought that Resource 2 had improved in comparison to the original version.

## Discussion

Through the use of a checklist that was based on assessing the suitability of health resources, this study resulted in improved SAM scores for resources that were created by RDs for RDs to enhance their knowledge on sugars and health. This, along with others, highlights the importance of appropriately engaging the proper end-users or seeking expert opinions when creating resources that are intended for a specific population. The SAM tool was selected because it is a widely used, validated framework for evaluating the suitability of health education materials, offering a structured approach to assess key elements such as content clarity, literacy demand, and visual design.

In this case, this study focused on Canadian RDs. Our methods are supported by others: Williams et al. used SAM to assess and improve patient-education materials in ophthalmology using evidence-based recommendations. Following the refinements, two glaucoma specialists scored the materials independently using SAM (Williams et al., 2016). The readability score improved from a tenth-grade reading level to a sixth-grade reading level and the mean SAM score of the materials improved from 60 ± 7% (adequate) to 88 ± 4% (superior) (Williams et al., 2016). Another study had health education specialists and educational technologists use SAM to evaluate the suitability of adolescent educational material in preventing hookah smoking in adolescents (Sadeghi et al., 2019). After refinement, the modified materials were given to 10 young adolescents for reassessment of their suitability. All materials improved in their SAM scores after being tailored based on the experts’ initial ratings (Sadeghi et al., 2019).

Specific to this study, the improved SAM scores through the evolution of the resources could be attributed to the use of our checklist, which was refined to incorporate questions and practical information in an easy-to-read manner aligned with SAM. For example, Resource 2 showed significant improvements for “Summary/Review” (*p* = 0.031) likely attributed to the checklist's emphasis on including summaries. Williams et al. reported similar improvements to this variable after the researchers only included crucial content, identified action steps, and only used words common to individuals without medical training (Williams et al., 2016). In this study, both resources improved in Survey 3 because the content focused more on dietetic practice, as opposed to general nutrition information.

Another example worth highlighting is the “Quick Glance” variable within Graphic Material, which significantly improved (*p* = 0.009) for Resource 2. Similarly, Types of Illustrations/Images significantly improved from *Survey* 1 to 3. The checklist's four recommendations on the Graphic Material (e.g., to incorporate a high contrast between paper and type, to use bright and fun colours, and to ensure that graphics appear professional) appear to have led to this rise in the SAM score.

Similarly, Cultural Images & Examples showed a major improvement in Resource 2 from *Survey 1* to *Survey 3* (*p* < 0.0001), which can be attributed to the checklist ensuring images are culturally appropriate and “match” the focus of the resource. Williams et al. achieved similar improvements in this variable by implementing the recommendation to “make certain content is appropriate for the age and culture of the target audience” (Williams et al., 2016).

It is acknowledged that the variable “Interaction” remained relatively low in their SAM scores compared to other variables. The SAM criteria for the variable of Interaction suggests the inclusion of such problems or quizzes, which were not included in our Resources. Finally, the proportion of “superior” ratings for Reading Grade Level in both resources started high, decreased in *Survey 2*, and increased again in *Survey 3*. These changes may be a result of participants being uncertain of the target audience of the education resources.

This study's limitation includes the fact that only English-speaking RDs were surveyed, which lacked the perspective of other-language-speaking individuals. The time between surveys was also long, therefore RDs may not have remembered the previous versions as clearly, which may have impacted the ratings. We did not ask RDs if they had previous experiences with SAM, which could have biased our results. Dietitians were surveyed on their views of the resources related to sugars and health, therefore the interpretation of our findings is limited to the specific topic area. Expanding the scope of application to a wide range of nutrition topics is warranted. Our sample size was too small to conduct internal consistency or reliability testing. Finally, while SAM is a widely used tool for evaluating health education materials, it has limitations, including the subjective nature of its scoring. It was also developed for print-based resources and may not fully reflect how people access health information today, particularly online. Additionally, while this study focused on dietitians, future work should include testing with end-users, as their understanding and needs may differ significantly from those of health professionals.

This study reinforces the importance of engaging with experts or end-users when developing educational resources. Through the use of our checklist, our study highlights the importance of choosing appropriate colours and graphics when developing or refining tools as these small details can improve overall suitability scores. Using the SAM checklist, RDs were able to revise the patient education materials to align with SAM-specific criteria, ensuring the nutrition education resources are suitable for their intended audience. Future studies should use our checklist to assess if other nutrition or health education resources improve SAM scores after their use.

## Supplemental Material

sj-pdf-1-nah-10.1177_02601060251365357 - Supplemental material for Enhancing nutrition education resources through the development and refinement of a checklist using the suitability assessment of materials (SAM) Supplemental material, sj-pdf-1-nah-10.1177_02601060251365357 for Enhancing nutrition education resources through the development and refinement of a checklist using the suitability assessment of materials (SAM) by Oliver Sage, Flora Wang, Chiara DiAngelo, Sandra Marsden, Claudia Faustini, Shannan Grant and Tamara R Cohen in Nutrition and Health

sj-pdf-2-nah-10.1177_02601060251365357 - Supplemental material for Enhancing nutrition education resources through the development and refinement of a checklist using the suitability assessment of materials (SAM) Supplemental material, sj-pdf-2-nah-10.1177_02601060251365357 for Enhancing nutrition education resources through the development and refinement of a checklist using the suitability assessment of materials (SAM) by Oliver Sage, Flora Wang, Chiara DiAngelo, Sandra Marsden, Claudia Faustini, Shannan Grant and Tamara R Cohen in Nutrition and Health

sj-docx-3-nah-10.1177_02601060251365357 - Supplemental material for Enhancing nutrition education resources through the development and refinement of a checklist using the suitability assessment of materials (SAM) Supplemental material, sj-docx-3-nah-10.1177_02601060251365357 for Enhancing nutrition education resources through the development and refinement of a checklist using the suitability assessment of materials (SAM) by Oliver Sage, Flora Wang, Chiara DiAngelo, Sandra Marsden, Claudia Faustini, Shannan Grant and Tamara R Cohen in Nutrition and Health

## References

[bibr1-02601060251365357] Anderson J Manias E Kusljic S , et al. (2014) Testing the validity, reliability and utility of the Self-Administration of Medication (SAM) tool in patients undergoing rehabilitation. Research in Social & Administrative Pharmacy 10(1): 204–216. 23735813 10.1016/j.sapharm.2013.04.013

[bibr2-02601060251365357] Doak CC Doak L Root J (1996) Teaching Patients with Low Literacy Skills, 2nd ed. Philadelphia: J.B. Lippincott Company.

[bibr3-02601060251365357] Sadeghi R Mazloomy Mahmoodabad SS Fallahzadeh H , et al. (2019) Predictive factors for preventing hookah smoking and health promotion among young people based on the protection motivation theory. Journal of Education and Health Promotion 8: 69. 31867354 10.4103/jehp.jehp_78_19 PMC6796312

[bibr4-02601060251365357] Smith F Carlsson E Kokkinakis D , et al. (2014) Readability, suitability and comprehensibility in patient education materials for Swedish patients with colorectal cancer undergoing elective surgery: A mixed method design. Patient Education and Counseling 94(2): 202–209. 24290242 10.1016/j.pec.2013.10.009

[bibr5-02601060251365357] Williams AM Muir KW Rosdahl JA (2016) Readability of patient education materials in ophthalmology: A single-institution study and systematic review. BMC Ophthalmology 16: 33. 27487960 10.1186/s12886-016-0315-0 PMC4973096

