# Supplementary material for: Enhancing nutrition education resources through the development and refinement of a checklist using the suitability assessment of materials (SAM)
Source: Nutr Health. 2025 Aug 17;32(1):31–40. doi: 10.1177/02601060251365357 (PMC12982570; doi:10.1177/02601060251365357)

Supplemental Figure 1. Resources on sugar and health: **A**) **Resource 1.** Effect of fructose-containing sugars on metabolic disease risk factors (Versions 1, 2 and 3); **B) Resource 2**. Uncover the truth about sugar: sources of sucrose (Version 1, 2 and 3).

A.

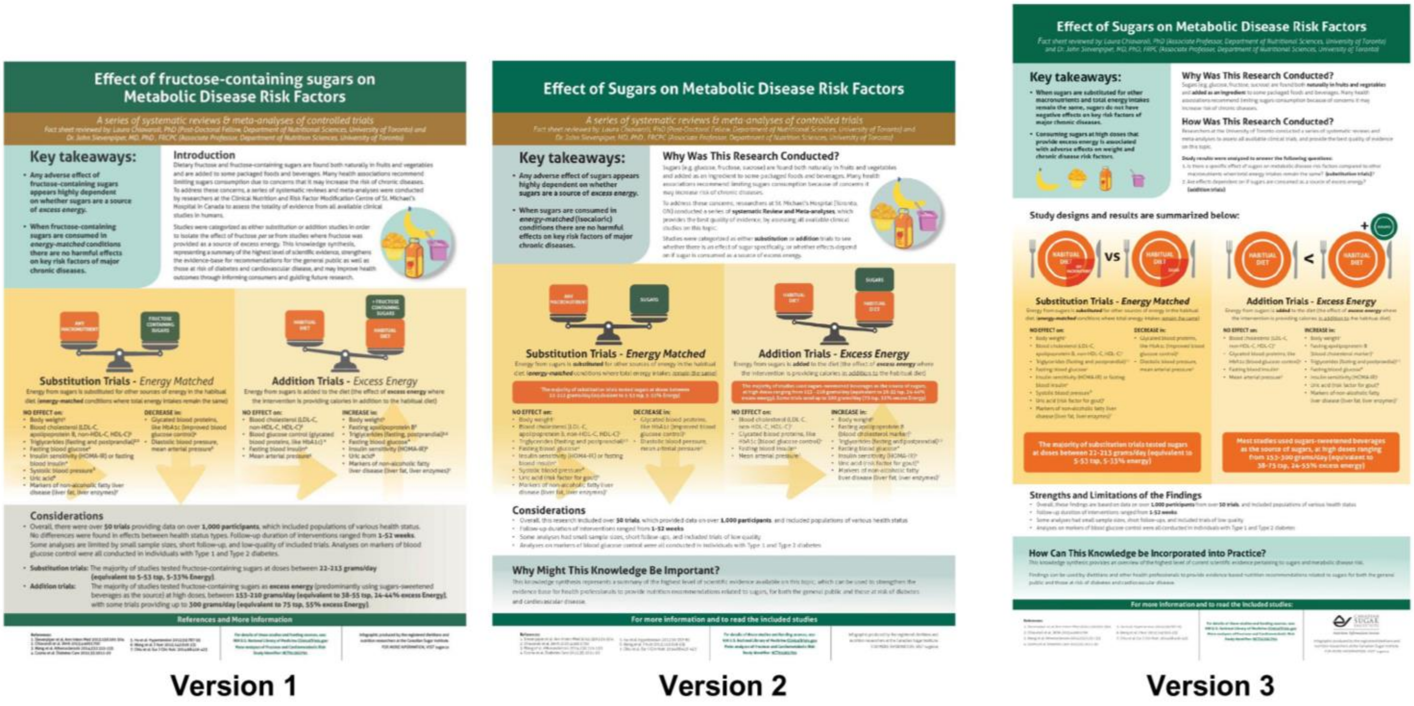

B.

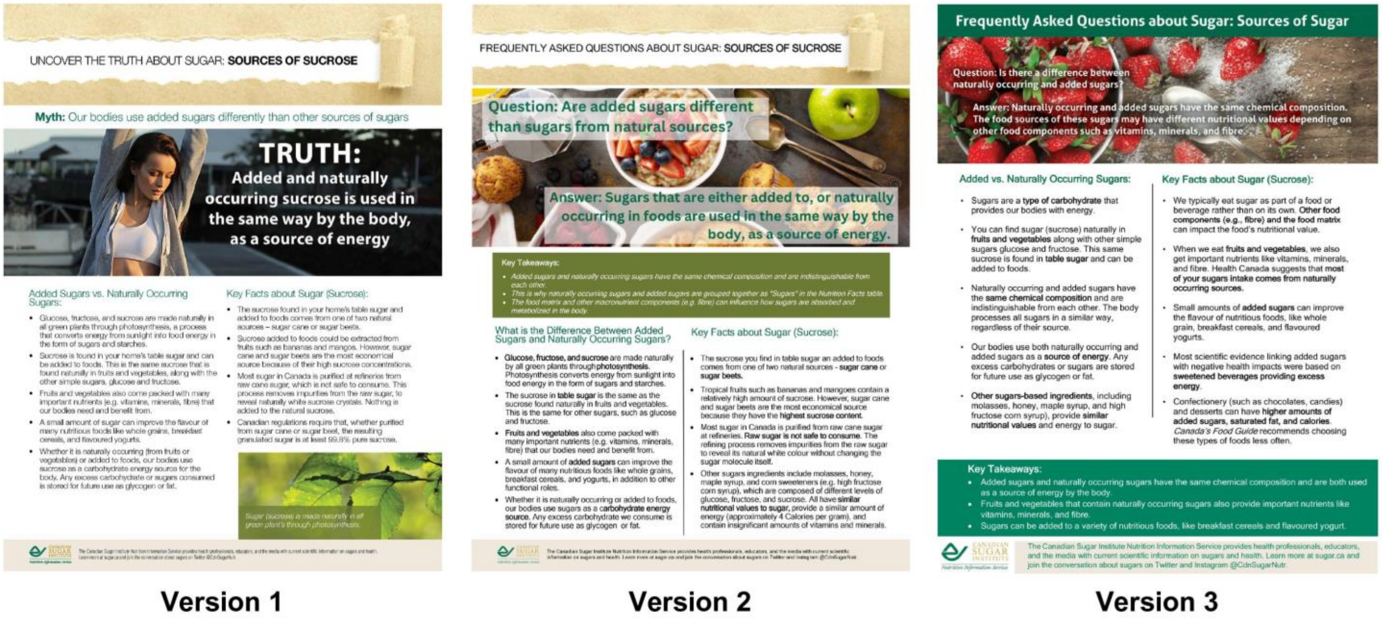

Supplement: sj-pdf-1-nah-10.1177_02601060251365357 - Supplemental material for Enhancing nutrition education resources through the development and refinement of a checklist using the suitability assessment of materials (SAM) [file sj-pdf-1-nah-10.1177_02601060251365357.pdf]
