# Supplementary material for: Enhancing nutrition education resources through the development and refinement of a checklist using the suitability assessment of materials (SAM)
Source: Nutr Health. 2025 Aug 17;32(1):31–40. doi: 10.1177/02601060251365357 (PMC12982570; doi:10.1177/02601060251365357)
Supplement: sj-pdf-2-nah-10.1177_02601060251365357 - Supplemental material for Enhancing nutrition education resources through the development and refinement of a checklist using the suitability assessment of materials (SAM) [file sj-pdf-2-nah-10.1177_02601060251365357.pdf]

Supplemental Figure 2: Version 1.0 of the Checklist

Nutrition education resource checklist

1. **Most important content should allow readers to increase and apply knowledge of topic**
   - Summaries are included and retell key messages in different words or examples
2. **Text should incorporate conversational style and active voice throughout**
   - Active voice makes it clear who is supposed to do what. It eliminates ambiguity about responsibilities.
   Examples:
   Take your vitamin everyday vs *Patients are advised to take their vitamin daily*
   We proposed new regulations vs *New regulations were proposed*
   - Conversational style means simple, short sentences, avoiding complicated words, and keeping a casual and easy-to-read writing style
3. **Simple and short sentences should be used whenever possible**
4. **Common words should be used whenever possible, technical words should be avoided**
5. **Technical, Concept, Category, Value Judgment words should always be explained**
   - Few or no words express general terms such as categories (Example: Use *milk* instead of *dairy products*) or value judgments (Example: Use *pain that does not go away in 5 minutes* instead of *excessive pain*).
6. **Context should be provided before introducing novel information**
7. **Overall look and feel of resource should attract attention, and clearly portray the purpose of the material**
   - High contrast between paper (background) and type (writing) attracts attention
8. **Images, graphics, and illustrations should be relevant to the content and purpose of the material**
9. **All or most illustrations and graphics should be accompanied by captions to "announce" or explain them**
10. **Layout should be consistent, should not be cluttered, and should incorporate visual cueing devices**
    - Sequence of information are consistent, making it easy to predict the flow of information
    - Visual cueing devices (boxes, arrows, shading) are used to direct attention to key content
11. **Interaction with reader is included by presenting problems or questions for reader response**
    - Problems or questions are presented for reader response
    - Knowledge-based questions (e.g., quiz)
12. **Complex topics are subdivided so that readers may experience small successes in understanding or problem-solving, leading to self-efficacy (confidence)**
13. **Images and examples are inclusive and present culture in positive ways**
    - I.e., stereotypical, oversimplified, and/or inaccurate representations of culture are avoided)
