# Supplementary material for: Enhancing nutrition education resources through the development and refinement of a checklist using the suitability assessment of materials (SAM)
Source: Nutr Health. 2025 Aug 17;32(1):31–40. doi: 10.1177/02601060251365357 (PMC12982570; doi:10.1177/02601060251365357)
Supplement: sj-docx-3-nah-10.1177_02601060251365357 - Supplemental material for Enhancing nutrition education resources through the development and refinement of a checklist using the suitability assessment of materials (SAM) [file sj-docx-3-nah-10.1177_02601060251365357.docx]

**Study Title:** **Enhancing Nutrition Education Resources through the Development and Refinement of a Checklist Using the Suitability Assessment of Materials (SAM)**

*These questions will give us insights into your perceptions of the nutrition education resource.

*(Show screenshots of validated tool/education resource)*

1. Purpose: It is important that readers readily understand the purpose of the materials. If they don’t clearly

perceive the purpose, they may miss the main points.

Check one:

- Superior
  - Purpose is explicitly stated in the title, cover illustration or introduction.
- Adequate
  - Purpose is not explicit. It is implied or multiple purposes are stated.
- Not suitable
  - No purpose is stated in the title, illustration, or introduction.

2. Content Topics: Adult learners usually want to solve their immediate health problem, rather than learn

medical facts. The content of most interest and use to readers is information that helps increase readers’ nutrition knowledge.

Check One:

- Superior
  - Material is conducive to leading readers to apply nutrition knowledge to their own lives
- Adequate
  - At least 40% of content topics focus on increasing nutrition knowledge.
- Not Suitable
  - Nearly no topics focus on increasing nutrition knowledge

3. Summary & Review: A review offers readers a chance to see the key points in other words, examples or

visuals and increases comprehension.

Check One:

- Superior
  - Summaries are included and retell key messages in different words or examples.
- Adequate
  - Some key topics are reviewed.
- Not Suitable
  - No summary or review is included.

4. Reading Grade Level: Text reading level is a critical factor in comprehension. Would you agree that the reading level is a suitable level for dietitians?

Check One:

- Strongly disagree
- Disagree
- Neutral
- Agree
- Strongly agree

5. Writing Style: Conversational style and active voice are easy to understand. Passive voice, embedded

information and long or multiple phrases slow reading and reduce comprehension. Example: Take your

vitamin every day is easier to understand than Patients are advised to take their vitamin daily.

Check One:

- Superior
  - 1)Conversational style and active voice are used throughout.
    2) Simple sentences are used extensively.
- Adequate
  - 1) About half the text uses conversational style, active voice.
    2) Less than half of sentences are complex with long phrases.
- Not Suitable
  - 1) Passive voice throughout.
    2) Over half of sentence have long or multiple phrases.

6. Sentence Construction: The context is given before new information. We learn new facts/behaviors more

quickly when told the context first. Example: To relieve pain (context), put heat on the sore spot (new

information).

Check One:

- Superior
  - Consistently provides context before presenting new information.
- Adequate
  - Provides context first about half the time.
- Not Suitable
  - Context is not provided.

7. Vocabulary: Common explicit words are used. (Example: Use doctor instead of physician). Few or no words

express general terms such as categories (Example: Use milk instead of dairy products) or value judgments

(Example: Use pain that does not go away in 5 minutes instead of excessive pain). Imagery words are used because

these are words that people can “see.” (Example: Use runny nose instead of excess mucus).

Check One:

- Superior
  - All three factors: 1) common words are used all the time.
    2) Technical, concept, category, value judgment words (CCVJ) are explained.
    3) Appropriate imagery words are used.
- Adequate
  - 1) Common words are used frequently.
    2) Technical CCVJ words are explained sometimes.
    3) Some jargon is used.
- Not Suitable
  - 1) Two or more factors
    2) Uncommon words are used frequently instead of common words.
    3) No explanation or examples are given for technical and CCVJ words.
    4) Extensive jargon.

8. Learning Enhanced by Advance Organizers (Road Signs): Headers or topic captions tell very briefly

what is coming next. These “road signs” make the text look less intimidating and prepare the reader’s

thought process to expect the announced topic.

Check One:

- Superior
  - Nearly all topics are preceded by an advance organizer (a statement that tells what is next).
- Adequate
  - About 50% of topics are preceded by advance organizers.
- Not Suitable
  - Few or no advance organizers are used.

9. Quick Glance: People do judge a book by its cover. The overall look and feel of a material is often the deciding factor in a reader’s attitude toward, and interest in, the materials.

Check One:

- Superior
  - The overall look and feel:
    1) Is friendly
    2) Attracts attention
    3) Clearly portrays the purpose of the materials
- Adequate
  - The overall look and feel has one or two of the superior criteria.
- Not Suitable
  - The overall look and feel has none of the superior criteria.

10. Type of Illustrations: Simple line drawings can promote realism without distracting details. Visuals are

accepted and remembered better when they portray what is familiar and easily recognized. Viewers may not

recognize the meaning of medical drawings or abstract symbols.

Check One:

- Superior
  - Both factors:
    1) Simple adult-appropriate line drawings/sketches are used.
    2) Illustrations are likely to be familiar to readers
- Adequate
  - One of the superior factors is missing.
- Not Suitable
  - None of the superior factors is present.

11. Relevance of Illustrations: Nonessential details such as room backgrounds, elaborate borders, unneeded

color can distract the viewer. The viewer’s eyes may be “captured” by these details. Illustrations should tell

key points visibly.

Check One:

- Superior
  - Illustrations present key messages visually so the reader can grasp the key ideas from illustrations alone. No distractions.
- Adequate
  - 1) Illustrations include some distractions.
    2) Insufficient use of illustrations.
- Not Suitable
  - No illustrations or an overload of illustrations.

12. Graphics: Lists, tables, charts, forms: Many readers do not understand the purpose of lists and charts.

Explanations or directions are essential.

Check One:

- Superior
  - Provides step-by-step directions with an example that will build self-efficacy (confidence).
- Adequate
  - “How to” directions are too brief for readers to understand and use the graphic without help.
- Not Suitable
  - Graphics are presented without explanation.

13. Captions are used to “announce” or explain graphics: Captions can quickly tell the reader what the

graphic is about and where to focus within the graphic. A graphic without a caption is usually an inferior

instruction and missed learning opportunity.

Check One:

- Superior
  - Explanatory captions with all or nearly all illustrations and graphics.
- Adequate
  - Brief captions are used for some graphics.
- Not Suitable
  - Captions are not used.

14. Typography: Type size and fonts can make text easy or difficult for readers at all skill levels. For example,

type in ALL CAPS slows everyone’s reading comprehension. When too many (6+) type fonts and sizes are

used on a page, the appearance becomes confusing and the focus uncertain.

Check One:

- Superior
  - At least 3 of the following 4 factors are present:
    1) Text type is in uppercase and lowercase.
    2) Type size is at least 12 point (This is 12-point type).
    3) Typographic cues (bold type, color, size of type).
    4) No ALL CAPS for long headlines and running text.
- Adequate
  - Two of the superior factors are present.
- Not Suitable
  - One or none of the superior factors are present.
  - Or 6 or more type styles/sizes are used on one page.

15. Layout: Layout has a substantial influence on the suitability of materials.

Check One:

- Superior
  - At least 5 of the following 8 factors are present:
    1) Illustrations are adjacent to the related text.
    2) Layout and sequence of information are consistent, making it easy to predict the flow of information.
    3) Visual cueing devices (boxes, arrows, shading) are used to direct attention to key content.
    4) Pages do not appear cluttered.
    5) Use of color supports and is not distracting to the message. Readers need not learn color codes to understand and use the message.
    6) Line length is 30 to 50 characters and spaces.
    7) There is high contrast between type and paper.
    8) Paper has a non-gloss or low-gloss surface.
- Adequate
  - At least 3 of the superior factors are present.
- Not Suitable
  - 1) Two or fewer of the superior factors are present.
    2) Looks uninviting or hard to read.

16. Subheadings and “chunking”: Few people can remember more than 7 independent items. For those with

low literacy skills the limit may be 3 or 5 items. Longer lists need to be partitioned into smaller chunks.

Check One:

- Superior
  - 1) Lists are grouped under descriptive subheadings.
    2) No more than 5 items are presented without a subheading.
- Adequate
  - No more than 7 items are presented without a subheading.
- Not Suitable
  - More than 7 items are presented without a subheading.

17. Interaction included in text and /or graphics: When a reader does something to reply to a question or

problem, chemical changes take place in the brain that enhance retention in long-term memory. Readers

should be asked to solve problems, make choices, demonstrate.

Check One:

- Superior
  - Problems or questions are presented for reader response.
- Adequate
  - Question & Answer format is used to discuss problems and solutions (passive interaction).
- Not Suitable
  - No interactive learning or stimulation is provided.

18. Desired behavior patterns are modeled or shown in specific terms: People often learn more readily when

specific, familiar instances are used rather than abstract or general concepts.

Check One:

- Superior
  - Instruction models specific behavior and skills. Example: nutrition information emphasizes changing eating patterns, shopping, cooking.
- Adequate
  - Information is a mix of technical and common language the reader may not easily interpret in terms of daily living. Example: High sugar, low nutrient value foods instead of No fuel foods
- Not Suitable
  - Information is presented in non-specific or category items such as food groups.

19. Motivation: People are motivated to learn when they believe tasks and behaviors are doable.

Check One:

- Superior
  - Complex topics are subdivided so that readers may experience small successes in understanding or problem solving, leading to self-efficacy (confidence).
- Adequate
  - Some topics are subdivided to improve readers’ confidence.
- Not Suitable
  - No partitioning is provided.

20. Cultural Match — Logic, Language, Experience (LLE): A valid measure of the cultural

appropriateness of material is how well its logic, language, and experience (inherent in the instruction) match

the LLE of the intended audience (not the reviewer). Example: Nutrition instruction is a poor cultural match if it

tells readers to eat vegetables that are rarely eaten by people in that culture and not sold in the reader’s

neighborhood.

Check One:

- Superior
  - Central concepts of the material appear to be culturally similar to the LLE of the target culture.
- Adequate
  - Significant match in LLE for 50% of central concepts.
- Not Suitable
  - Clearly a cultural mismatch in LLE.

21. Cultural Image and Examples: To be accepted, an instruction must present cultural images and examples

in realistic and positive ways.

Check One:

- Superior
  - Images and examples present culture in positive ways.
- Adequate
  - Neutral presentation of cultural images and foods.
- Not Suitable
  - Negative images such as exaggerated or caricatured cultural characteristics, actions, or examples.

22. Suitable for your population? Considering the socioeconomic and cultural backgrounds present in your

population and your review of the Beginnings Guides Curriculum, would you recommend Beginnings for your

program. Circle the number that shows the strength of your recommendation.

0 1 2 3 4 5 6 7 8 9 10

NO YES

Definitely not Recommended

recommended without reservation

**These questions will help us understand who you are as individuals.

23.What is your date of birth? (Month): _______ (Year): _______ Refuse to answer

24. What is your gender? Please specify_________ Refuse to answer

25. People living in Canada come from many different cultural and racial backgrounds. Are you...? (Please check one or more)

- White
- Aboriginal/ First Nations/ Métis
- South Asian (e.g., East Indian, Pakistani, Sri Lankan, etc.)
- Chinese
- Black
- Filipino
- Latin American
- Arab
- Southeast Asian (e.g., Vietnamese, Cambodian, Laotian, etc.)
- West Asian (e.g., Iranian, Afghan, etc.)
- Korean
- Japanese
- Other, please specify: _____________
- Refuse to answer

26. Where do you practice dietetics? (Choose all that apply)

- Alberta
- British Columbia
- Manitoba
- New Brunswick
- Newfoundland and Labrador
- Northwest Territories
- Nova Scotia
- Nunavut
- Ontario
- Prince Edward Island
- Quebec
- Saskatchewan
- Yukon
- Refuse to answer

27. To which professional regulatory body do you belong? (Choose all that apply)

- College of Dietitians of Alberta
- College of Dietitians of British Columbia
- College of Dietitians of Manitoba
- New Brunswick Association of Dietitians
- Newfoundland and Labrador College of Dietitians
- Nova Scotia Dietetic Association
- College of Dietitians of Ontario
- College of Dietitians of Prince Edward Island
- Ordre professionnel des diététistes du Québec
- Saskatchewan Dietitians Association

28. In which area(s) do you practice dietetics? (Choose all that apply)

- Private practice
- Acute hospital
- Outpatient care
- Long term care
- Population and Public Health
- Food Service Management
- Other, please specify: _____________
- Refuse to answer

29. Which types of clients do you work with? (Choose all that apply)

- Eating disorders
- Weight loss
- Oncology patients
- CVD
- Nutrition support (parenteral, enteral, supplementation)
- Diabetes (type 1 and/or 2)
- Gastroenterology
- Hepatology
- Surgery
- Pediatric population
- Other, please specify: _____________
- Refuse to answer

30. How many years have you been practicing as a dietitian?

- ≤2 years
- 2-5 years
- 5-10 years
- 10-15 years
- 15-20 years
- 20-30 years
- 30+ years
- Refuse to answer
